# Modifying Health Behavior to Prevent Cardiovascular Diseases: A Nationwide Survey among German Primary Care Physicians

**DOI:** 10.3390/ijerph110404218

**Published:** 2014-04-15

**Authors:** Sven Schneider, Katharina Diehl, Christina Bock, Raphael M. Herr, Manfred Mayer, Tatiana Görig

**Affiliations:** 1 Mannheim Institute of Public Health, Social and Preventive Medicine, Medical Faculty Mannheim, Heidelberg University, Ludolf-Krehl-Str. 7-11, D-68167 Mannheim, Germany; E-Mails: katharina.diehl@medma.uni-heidelberg.de (K.D.); christina.bock@medma.uni-heidelberg.de (C.B.); raphael.herr@medma.uni-heidelberg.de (R.M.H.); tatiana.goerig@medma.uni-heidelberg.de (T.G.); 2 Internistic Group Practice Dr. med. Manfred Mayer und Dr. med. Angela Schmid, Max-Joseph-Str. 1, D-68167 Mannheim, Germany; E-Mail: mail@manfred-mayer.de; 3 Physician Network, Ärztenetz Qu@linet e.V., Liebfrauenstr. 21, D-68259 Mannheim, Germany

**Keywords:** primary care physician, general practitioner, counseling, prevention, health, health behavior, preventive measures, cardiovascular diseases

## Abstract

Cardiovascular diseases (CVD) are a major public health concern as they are the leading cause of death in developed countries. Primary care is considered to be the ideal setting for CVD prevention. Therefore, more than 4,000 German primary care physicians (PCPs) were asked about their attitudes towards and their activities regarding the prevention of CVD in the nationwide ÄSP-kardio Study. The focus of the study was on health behavior modification. Two thirds of the participating PCPs stated that they routinely provided brief inventions to assist patients in reducing both their tobacco (72%) and alcohol (61%) consumption, to encourage them to increase their levels of physical activity (72%), and to assist them in adjusting to a more healthy diet (66%), and in achieving a healthy body weight (69%). However, only between 23% (quitting smoking) and 49% (diet modification) of PCPs felt that they had been successful in helping patients modify their lifestyles. Insufficient reimbursement, cultural diversity and a lack of time were reported to be the most problematic barriers to successful intervention in the primary care setting. Despite these obstacles, the majority of German PCPs was engaged in prevention and health behavior intervention to reduce the incidence and progression of CVD.

## 1. Introduction

Cardiovascular disease (CVD) is a leading cause of death worldwide, causing more than 13 million mortalities annually [1]. More than 340,000 people died as a result of CVD in Germany alone in 2011 [2]. This represents 40.2% of all deaths in Germany that year, and similarly high percentages of CVD-related mortalities have been recorded in other industrialized nations. Due to its chronic nature, CVD causes substantial treatment costs, which in turn put the healthcare systems in Germany and other comparable countries under immense financial pressure [3]. This situation highlights the need for increased levels of prevention to effectively lower not only the occurrence of CVD itself, but also to reduce the drain the disease’s treatment has on national healthcare budgets.

In contrast to age, gender and genetic predisposition, modifiable CVD risk factors can be positively affected by instigating appropriate intervention measures and thus lowering a patient’s risk of developing CVD. An individual’s CVD risk level is largely associated with his or her lifestyle [4,5]. The most relevant lifestyle related risk factors include tobacco and alcohol consumption, low levels of physical activity, unhealthy eating habits and obesity.

Smoking in particular has been identified as one of the central risk factors in the pathogenesis of CVD; even low levels of regular tobacco consumption increase a patient’s risk of developing CVD [6]. However, if a patient stops smoking early enough, his or her mortality risk level will eventually return to that of a non-smoker [7]. Additionally, quitting smoking also leads to an improved prognosis for patients already diagnosed with CVD [8].

A J-shaped association has been shown between alcohol consumption and CVD [9,10]. However, whether moderate alcohol consumption leads to a reduction in the risk of CVD is the subject of an ongoing controversial debate [11,12]. Due to the negative health and social effects of excessive consumption, alcohol cannot be recommended as a CVD prevention measure. On the contrary, alcohol consumption can have an adverse effect on a patient’s cardiovascular risk [13].

Approximately 30% of all cardiovascular disorders develop due to a lack of physical activity [5]. Numerous cohort studies have shown that increased physical activity is associated with a significant reduction in CVD risk among both men and women e.g., [14,15]; a dose-response relationship has been identified which associates increased levels of physical activity with a corresponding rise in health benefit [16].

Additionally, previous studies have also shown associations between the consumption of numerous foodstuffs and the development of CVD. In particular, saturated fats and foods with high glycemic indices are associated with an increased risk of cardiovascular disease. In contrast to this, the consumption of vegetables and nuts, as well as Mediterranean dietary patterns have a protective effect on the cardiovascular system [17]. Furthermore, excess body weight and adiposity, both of which have reached epidemic proportions in the industrialized nations, also increase the likelihood of CVD [18]. In this context it is well known that, due to this is endocrinal activity, visceral body fat in particular increases the risk of CVD [19].

The high burden of disease caused by lifestyle risk factors also means that corresponding changes in lifestyle can effectively contribute to CVD prevention. PCPs’ practices offer the ideal setting for intervention measures aimed at modifying patients’ lifestyle habits for the purpose of primary, secondary and tertiary cardiovascular disease prevention. Nine out of ten adults in Germany visit a physician at least once a year [20]. Depending on the patients’ age, up to 80% of these visits are to primary care physicians (PCPs) [21]. This level of regular contact with patients allows PCPs to repeatedly intervene and instigate CVD prevention measures which can be adapted to suit each individual patient’s needs. Thus PCPs play a central role in the instigation and implementation of individualized CVD prevention measures.

The German Society of General Practice and Family Medicine (Deutsche Gesellschaft für Allgemein- und Familienmedizin, DEGAM) defines CVD prevention as one of the principal duties of a PCP, despite the fact that prevention measures such as lifestyle counseling are thus far under-represented in the German healthcare remuneration system [22]. In Germany, about 90% of all citizens have a state health insurance plan. The remaining 10% have private, and thus more comprehensive health insurance. The state health insurance guarantees to provide each insured person with the same treatment. This national homogeneous health insurance system reimburses PCPs for the treatment of CVD but not for primary prevention measures against the development of CVD (e.g., lifestyle counseling, motivational interviewing, and nicotine replacement therapy). This is why CVD risk factors and lifestyle prevention parameters are not systematically and consistently recorded by PCPs. And this despite the fact that the conditions for the promotion of primary prevention are very promising: The advantages of preventive measures provided by PCPs are based above all on the stable and usually well-established relationship between a general practitioner and his or her patients. This relationship ensures that the PCP not only knows the individual risk of each patient, but also his or her individual abilities, resources, strengths and weaknesses. This kind of background information is especially important when implementing measures to modify a patient’s lifestyle.

Randomized-controlled studies have already shown that lifestyle interventions by PCPs are effective in significantly reducing both cardiovascular risk and the progression of CVD [4]. For example, a recently published randomized-controlled study from Belgium showed that something as simple as a medical assessment (a screening of the above mentioned risk factors and a follow-up) by a general practitioner can lead to a significant decrease of the overall cardiovascular risk—and that even independent of an additionally conducted tailored coaching [23]. Therefore, many national and international medical associations are calling for the integration of lifestyle modification measures into routine PCP-patient discussions [22,24,25,26,27].

So far relatively few studies have been carried out on the practice of lifestyle interventions by PCPs [28]. To date no representative and detailed data on lifestyle interventions offered by PCPs in Germany has been published. Therefore, it is unclear how prevention strategies are being initiated in German medical practices and which possible obstacles to lifestyle-based prevention measures have to be overcome. The study presented in this paper aims to shine some light into the “black box” of the PCP’s practice. The concrete goals of the study were: (1) to describe the attitudes of German PCPs towards health promotion and CVD prevention; (2) to detail the lifestyle-based prevention measures currently being implemented in a PCP’s day-to-day routine; and (3) to identify possible obstacles to the implementation of such lifestyle-based prevention measures.

## 2. Methods

### 2.1. Sample and Data Collection

The PCPs (here: general practitioners, medical practitioners and internists working as general practitioners) who were invited to take part in the study were sent a questionnaire between October 2011 and March 2012. The questionnaire included questions regarding PCPs’ personal attitudes towards health promotion and CVD prevention, the measures with which they regularly intervene to help patients change their lifestyles and which partners they cooperate with when implementing CVD prevention measures. The questionnaire consisted of field-tested questions used in previous studies. The selection of the questions was based on a systematic review that had been conducted to this end beforehand [28]. Furthermore, before determining its final version, the questionnaire was subject to an evaluation by an external commission of experts (Delphi-Methode) and to a face-to-face validation by means of 1-hour cognitive interviews with ten PCPs. In addition to that it was tested in the framework of a regional pilot study which included a sample of 260 PCPs [29,30].

The sample of PCPs included in the final study was selected using random stratification by sex, medical specialty and federal state from the database provided by ArztData GmbH, Hamburg, Germany. The study had been announced in several relevant medical journals before the questionnaires were mailed to the PCPs. Subsequently, the PCPs were sent a letter inviting them to take part in the study. One week after they had received this letter they were then sent the standardized questionnaire, together with a cover letter containing further information on the study’s aims, information on data protection and a self-addressed, pre-stamped envelope. One week later all PCPs were then sent a reminder postcard. After a further four weeks had passed, a second round of questionnaires was sent out to those PCPs who had not yet responded to the first delivery. Additionally, the PCPs were given the alternative of filling in an online questionnaire. The study design and procedures were approved by the ethic committee of the Medical Faculty Mannheim, Heidelberg University (2008-272E-MA).

A total of 4074 PCPs participated in the survey. The response rate was 33.9% [31]. The participants were paid €20 as reimbursement for the time they needed to fill in the questionnaire (about 15 to 20 min).

### 2.2. Study Variables

This paper presents the main results of the ÄSP-kardio Study. The attitudes, self-competence, and self-efficacy of PCPs towards health promotion and prevention were drawn from the studies by Steptoe *et al.* and Hulsher *et al.* [32,33]. The level of PCPs’ agreement with these statements was rated on a 4-point scale (completely agree, slightly agree, slightly disagree, completely disagree). PCPs also had the opportunity to rate the perceived importance of smoking cessation, reduction of alcohol consumption, promotion of physical activity, a healthy diet and achieving a healthy weight for the prevention of CVD (very important, quite important, rather not important, not important at all). We also gathered information on the PCPs self-rated competence in providing intervention measures to alter the above-mentioned lifestyle factors and the perceived success of these interventions (very high, quite high, rather low, very low).

Additionally, details on individuals and institutions with which PCPs cooperated when implementing CVD prevention measures were gathered. Furthermore, obstacles in the PCPs’ daily routines that prevent them from implementing lifestyle-based intervention measures were explored by asking PCPs to rate their agreement with several statements (e.g., “I do not have time to offer lifestyle advice” or “I lack the training to offer lifestyle advice”) using another 4-point Likert scale (completely agree, slightly agree, slightly disagree, completely disagree).

The frequency of provision of lifestyle advice was examined by asking PCPs how many of their adult patients were offered a brief intervention to assist with quitting smoking, reducing alcohol consumption, increasing physical activity, or achieving a healthy diet. Based on a definition from Walsh *et al.* [34], a PCP was considered to be routinely implementing lifestyle-based intervention measures when the majority of his or her patients (more than 50%) were offered such measures.

### 2.3. Descriptive Statistics

We used descriptive methods to analyze our data. The percentages calculated (%) always refer to valid cases. The mean (M) and the standard deviation (SD) were calculated for normally distributed variables. All analyses were performed using IBM SPSS Statistics, Version 21.0 (Armonk, NY, USA).

## 3. Results

The majority of PCPs included in the sample were general practitioners. One quarter were internists working as general practitioners (Table 1). Six out of ten PCPs included in the sample were male. The participants were on average 52 years old and had been resident for approximately 13 years. More than half of the PCPs had their own practice. More than 83% of the practices were located in urban or semi-urban areas. There were no significant differences between the study sample and the overall population of German PCPs regarding the distribution of sex, medical specialty and federal region. The participating PCPs reported seeing an average of 224 patients per week. According to the data provided by the PCPs, four out of ten of their patients were male, half were over 65 and 30% suffered from a manifest CVD. The data collected from the participating PCPs is detailed in Table 1.

The participating PCPs showed a generally positive attitude towards health promotion and CVD prevention in a general practice. Following descriptions refer to the percentage of PCPs who completely or slightly agreed with the statements (Table 2). Almost all PCPs (60.2% + 35.9% = 96.1%) saw themselves as health advisors. Concerning their self-reported competence eight out of ten PCPs felt they were adequately qualified to provide lifestyle advice (23.3% + 56.1% = 79.4%). Three quarters of the participants felt they could generally offer their patients a great deal in lifestyle advice (23.6% + 50.1% = 73.7%). However, only seven out of ten PCPs were certain that they could successfully motivate their patients to change their lifestyles for the better (8.8% + 62.1% = 70.9%).

**Table 1:** Characteristics of PCPs (*n* = 4074) included in the ÄSP-kardio Study, Germany.

	*n* (%) or M ± SD	
Sex		
Male	2391	(60.0%)
Female	1593	(40.0%)
Age (years)	51.5	±8.7
Years since residency	13.3	±9.2
Medical specialty		
General practitioners	2792	(68.7%)
Medical practitioners	314	(7.7%)
Internists	961	(23.6%)
Patient contacts per week	223.7	±130.0
Type of practice		
Solo practice	2114	(52.1%)
Group practice	1616	(39.8%)
Practice Sharing	311	(7.7%)
Location of practice		
Urban area	2020	(49.7%)
Semi-urban area	1382	(34.0%)
Rural area	664	(16.3%)

*n*: Number of PCPs (Total: *n* = 4074). % refer to valid cases. M: mean. SD: standard deviation.

**Table 2:** Attitudes, self-competence, and self-efficacy of PCPs towards health promotion and CVD prevention (Agreement with statements in % of PCPs).

	Completely agree	Slightly agree	Slightly disagree	Completely disagree
It is my duty not only to treat illnesses, but also to act as a health advisor.	60.2%	35.9%	3.0%	1.0%
I can offer my patients a great deal in lifestyle advice.	23.6%	50.1%	24.0%	2.4%
I am well qualified to provide advice regarding a healthy lifestyle.	23.3%	56.1%	19.1%	1.6%
I am one of the most important factors influencing the healthy lifestyle of my patients.	14.6%	54.0%	29.3%	2.1%
I can successfully motivate my patients to live healthier.	8.8%	62.1%	28.3%	0.7%

% refer to valid cases (Total: *n* = 4074). Due to rounding the percentages listed do not always add up to 100%.

**Figure 1:**
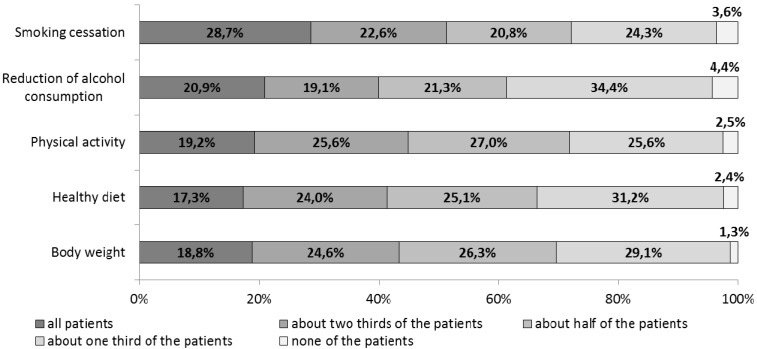
Provision of lifestyle-based intervention measures in a primary care setting, Germany (% of PCPs).

On average, the participating PCPs dedicated 36% of their working time to the prevention and treatment of CVD. As shown in Figure 1, around two thirds of them routinely provided (*i.e.*, to more than 50% of the patients) brief interventions to help patients quit smoking (28.7% + 22.6% + 20.8% = 72.1%), reduce their alcohol consumption (20.9% + 19.1% + 21.3% = 61.3%), increase their physical activity levels (19.2% + 25.6% + 27.0% = 71.8%), change to a healthy diet (17.3% + 24.0% + 25.1% = 66.4%) or achieve a healthy body weight (18.8% + 24.6% + 26.3% = 69.7%).

% refer to valid cases (Total: *n* = 4074).

**Figure 2:**
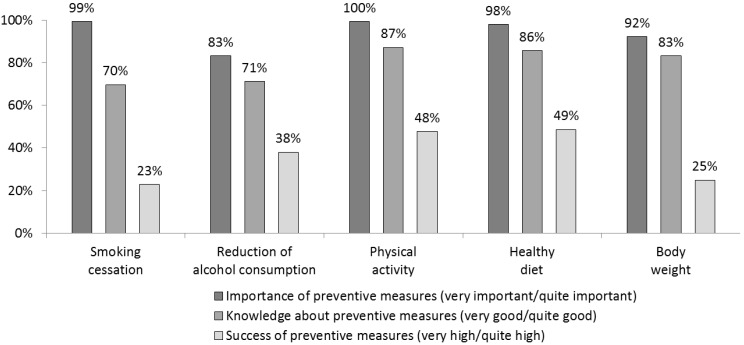
Importance, knowledge levels and success rates of preventative measures (% of PCPs).

A large majority of PCPs considered providing patients with advice on lifestyle-based CVD risk factors to be of great importance. In comparison to this, only between 70% (2830/4058; measures to assist patients with quitting smoking) and 87% (3528/4045; measures to increase patients’ physical activity) of PCPs believed they are well qualified to provide such advice (Figure 2). This difference is even more pronounced when it comes to the perceived success of these intervention measures; whereas around half of the PCPs felt they had been successful in motivating their patients to improve their diet (49%) and to increase their level of physical activity (48%), only 23% believed they had been able to convince their patients to quit smoking.

**Figure 3:**
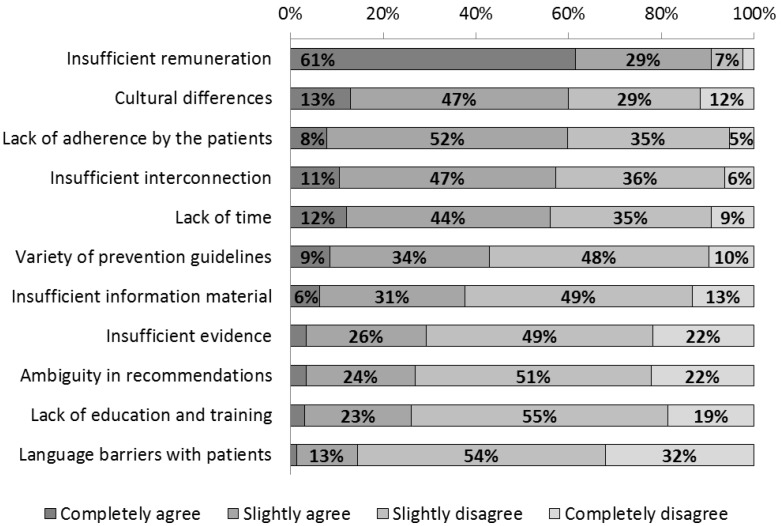
Obstacles to the provision of lifestyle advice in a general practitioner setting (% of PCPs).

PCPs named the lack of appropriate remuneration as the main obstacle to providing their patients with lifestyle advice (61% + 29% = 90%; Figure 3). Other relevant obstacles mentioned were different cultural perceptions of what constitutes a healthy lifestyle (13% + 47% = 60%), patients not adhering to lifestyle changes agreed upon (8% + 52% = 60%), insufficient opportunities to collaborate with other doctors and service providers (11% + 47% = 58%) and a lack of time during office hours (12% + 44% = 56%).

PCPs most regularly cooperated with their medical colleagues when implementing CVD prevention measures (87%). Approximately two thirds of the participating PCPs reported working with diabetes (68%) and ambulant cardiac groups (66%). Similar numbers of PCPs had established cooperative arrangements with alcohol rehabilitation centers (56%), sporting clubs (53%) and nutritionists (52%). Health insurance companies and smoking cessation support services were named as cooperation partners by 34% and 25% of PCPs respectively.

## 4. Discussion

It was shown that, despite numerous obstacles, German PCPs invest a considerable amount of their time in implementing CVD prevention measures. However, the provision of brief inventions to assist patients in changing their lifestyles was not always guaranteed. PCPs routinely provided interventions to assist patients with quitting smoking and increasing their levels of physical activity most often. The next most common interventions were aimed at helping patients achieve a healthy body weight, modify their diet and reduce their alcohol consumption. The participating PCPs generally had a positive attitude towards health promotion and believed that lifestyle modification played an important role in the prevention of CVD. However, not all PCPs felt well qualified to provide lifestyle advice and less than half of the PCPs asked believed they had been able to successfully motivate patients to change their lifestyles.

Due to specific characteristics of the German healthcare system and methodological differences in previous studies (e.g., some of them collected data using direct observation or patients’ responses), it is difficult to compare the results of this study directly with similar surveys previously published. Some parallels, however, are evident. For example, in comparison to other international studies, we could identify similar frequencies of interventions aimed at increasing patients’ physical activity levels (72% *vs.* 41% to 77%) [35,36,37], but less frequent interventions to motivate patients to change their diet (66% *vs.* 87% to 81%) [35,38]. In accordance with previous research, a difference between PCP’s knowledge levels and the self-rated effectiveness of the lifestyle interventions they provided could be identified. This disparity was especially evident concerning smoking cessation: whereas seven out of ten PCPs felt well qualified to provide advice on quitting smoking, two out of ten did believe they had been successful in motivating their patients to quit. Two studies from the USA reported finding similar results on tobacco cessation intervention measures (62% to 91% *vs.* 14% to 40%), as well as on several other lifestyle aspects looked at here [39,40].

The ÄSP-kardio Study identified numerous obstacles to the provision of lifestyle-based interventions by PCPs. The relative importance of several obstacles reported by PCPs in this survey were similar to those previously published; for example, lack of time (56% *vs.* 23% to 94%) [41,42,43,44,45] and cultural differences between doctor and patient (60% *vs.* 61%) [45]. In comparison, less agreement was found concerning the lack of availability of information material for patients (37% *vs.* 45% to 69%), and insufficient qualification for the provision of lifestyle advice (25% *vs.* 48% to 83%) [41,42,45], which indicates that German PCPs have adequate access to printed information material and have been provided with suitable medical training to provide lifestyle advice. However, in comparison to studies from other countries, it was found that PCPs in Germany more often name low patients’ adherence to the above-mentioned lifestyle changes (70% *vs.* 17% to 52%) [42,43,44] and insufficient remuneration as obstacles to the provision of lifestyle advice during office hours (90% *vs.* 30%) [43,44].

Comparing the data detailed here with that from a study on general practitioners carried out in six European countries enables the evaluation of the provision of advice in Germany with regard to the length of medical consultations: The approximately 2800 consultations filmed as part of the EUROCOM study showed that German PCPs allocated by far the least time to each patient (7.6 min) [46]. To help alleviate the time pressure German PCPs experience during office hours and thus improve the provision of lifestyle-based interventions, measures such as sharing of counseling activities and/or the use of additional resources should be considered. This could be achieved, for example, by employing the aid of practice nurses or other healthcare service providers [47]. It has previously been shown that additional training for such members of the medical profession can dramatically improve the provision of lifestyle advice to patients [48,49]. However, the obstacle “lack of time” was only ranked fifth on the list of factors the PCPs believed were preventing them from implementing adequate CVD prevention measures. According to the participating PCPs, insufficient remuneration is by far the most important barrier to the provision of lifestyle advice.

These findings suggest that changes must first be made within the German healthcare system to increase both the time and financial recourses available to PCPs to improve the provision of such lifestyle intervention measures. The fact that in Germany preventive actions of CVD are only reimbursed in case of secondary prevention was already discussed in the introduction. Despite recently introduced reforms, this fundamental problem could not yet be resolved: Health political discussions about the impact of chronic diseases have currently achieved reforms regarding the remuneration for more time-consuming patient consultations: Since 1 October 2013, German PCPs can charge comprehensive consultations for patients who are already suffering from a disease as an individual service. The prerequisite is that the consultation must last ten minutes or longer and is conducted because of a diagnosed life-changing disease (e.g., diagnosis of a cardiovascular disease). PCPs will be reimbursed €9 per 10 min completed for such consultations. However, this applies only to patients already suffering from a disease.

When interpreting the data presented here, it is important to take limitations into consideration such as: (1) a social desirability bias; (2) scope for interpretation; (3) lack of information on the lifestyle of the participating PCPs; (4) non-responder effects; and (5) restricted generalizability to other nations.

(1) Instead of a survey of PCPs, other studies have chosen alternative methods such as a survey of patients or a direct observation study. However, these methods also present an increased risk of socially desirable responses and advisory behavior [50]. Furthermore, due to their high level of ethical responsibility and scientific education it can be assumed that doctors generally give valid responses.
(2) Additionally, the fact that several terms used in the questionnaires can be interpreted in various ways (for example the term “brief intervention”) must be taken into consideration when interpreting the data presented in this study. In particular, the term “perceived success” might be subject to various interpretations. At this point it needs to be underlined that measuring success objectively is hardly ever realizable in a short paper and pencil questionnaire. However, our cognitive in-depth interviews revealed that PCPs understood this question as intended and previous studies had made use of this measurement, too [39,45].
(3) Furthermore, we deliberately avoided asking the participating PCPs about their own lifestyles. The cognitive interviews carried out prior to the beginning of the main survey showed that PCPs often felt controlled by these sorts of questions. In order to reduce the risk of PCPs giving falsified responses or refusing to take part in the survey, we decided to forgo gathering this undoubtedly interesting, but nevertheless sensitive information.
(4) The response rate of 33.9% is comparable with other studies of German PCPs. Only studies with much smaller sample-sizes have reported higher response rates (54% with *n* = 1179 [51] and 48% with *n* = 657 [52]). Other German physicians’ surveys have recorded comparatively lower response rates (27% with *n* = 1863 [53], 23% with *n* = 1863 [54], 15% with *n* = 4090 [55], 7% with *n* = 370 [56], 6% with *n* = 20,000 [57] and 3% with *n* = 25,000 [58]). Additionally, the characteristics of the PCPs included in the ÄSP-kardio Study sample did not differ significantly from those of the general German PCP population.
(5) The aim of our manuscript was to describe the attitudes of German PCPs towards health promotion and CVD prevention. Therefore, we did not use methods for statistical interference. Since the health care systems differ widely in Europe and worldwide, our results cannot be generalized to other countries. However, as we showed before, our study sample was representative for Germany. Therefore, our results describe the opinion of German PCPs.

## 5. Conclusions

Based on information provided by German PCPs, the ÄSP-kardio Study has shown that systematic cardiovascular prevention measures have not yet become a standard part of primary medical care in Germany. Low intervention rates, together with high CVD prevalence in the German population indicate at best wasted prevention potential and at worst a healthcare deficit. PCPs seem to be in an ideal position to initiate lifestyle modifications, as they have regular contact with their patients and are often the first point of contact regarding health-related questions. Despite the fact that the participating doctors indicated having a positive attitude towards health promotion and CVD prevention, the majority of PCPs reported experiencing a variety of problems when attempting to provide lifestyle advice during office hours. These problems have to do both with the feeling that they were likely to be unsuccessful in their attempts to motivate patients to change their lifestyles, and with obstacles presented by German healthcare system. Although primary prevention is known to be economically more efficient than treating existing health problems, at the time of the present survey the German healthcare system did not provide sufficient incentives for primary cardiovascular prevention by general practitioners. The fact that German PCPs continue to invest a considerable proportion of their time in providing cardiovascular prevention measures despite this lack of incentives shows that they take their duty as health advisors and their responsibility towards their patients very seriously.
